# Rice-Buckwheat Gluten-Free Pasta: Effect of Processing Parameters on Quality Characteristics and Optimization of Extrusion-Cooking Process

**DOI:** 10.3390/foods8100496

**Published:** 2019-10-14

**Authors:** Abdallah Bouasla, Agnieszka Wójtowicz

**Affiliations:** 1 Laboratoire de Génie Agro-Alimentaire, Institut de la Nutrition, de l’Alimentation et des Technologies Agro-Alimentaires (INATAA), Université Frères Mentouri Constantine 1, 325 Route de Ain El Bey, Constantine 25017, Algeria; abdallah.bouasla@umc.edu.dz; 2 Department of Thermal Technology and Food Process Engineering, University of Life Sciences in Lublin, Głęboka 31, 20-612 Lublin, Poland

**Keywords:** gluten-free pasta, rice-buckwheat, extrusion-cooking, process optimization, Box-Behnken design, cooking properties, texture, microstructure

## Abstract

A new type of gluten-free pasta has been developed based on a rice-buckwheat mixture. The aim of the study was to investigate the effect of process parameters of moisture content (30, 33, and 36%), barrel temperature (80, 100, and 120 °C), and screw speed (60, 80, and 100 rpm) on cooking and textural properties of rice-buckwheat pasta produced by a single-screw extrusion-cooker. The process uses response surface methodology based on a Box-Behnken experimental design. Results showed that with regard to this rice-buckwheat pasta, raising moisture content of the raw materials increased cooking loss and stickiness, but decreased firmness, while increasing barrel temperature reduced cooking loss and stickiness, but increased hardness and firmness. Screw speed increase also affected positively hardness and firmness of the obtained products. Thus, optimal conditions (moisture content 30%, barrel temperature 120 °C, and screw speed 80 rpm) were established to produce good quality rice-buckwheat pasta. At this optimum, the pasta showed a compact and homogeneous inside microstructure. Furthermore, the pasta products exhibited low cooking loss (less than 6%), good hardness and firmness, with low stickiness and acceptable scores for all sensory attributes and for overall quality.

## 1. Introduction

Pasta is universally consumed and appreciated due to its palatability, easy preparation, inexpensiveness, and long shelf-life [1]. Durum wheat semolina is the most suitable raw material for pasta production [2]. However, for some individuals, gluten intake brings about celiac disease. This is one of the most common food intolerances. Indeed, studies show that the prevalence is increasing and reaching 1% in most parts of the world [3].

At present, a strict life-long gluten-free diet is the only effective therapy for this disease. As a result, gluten-free products have been developed, not only for celiac patients and those with a gluten-related disorder but also for the rest of population [3]. However, low nutritional quality and poor mouthfeel and flavor have been reported for many gluten-free foods [4]. Such effect could be justified partially by the fact that these products are rarely enriched [5].

Among raw materials, buckwheat (*Fagopyrum esculentum*) has certain nutritional and functional properties which are deficient or absent in gluten-free cereal grains (rice and corn) [6]. The interesting nutritional properties of buckwheat partly stem from the presence of many valuable compounds such as high-quality proteins, minerals, B vitamins, dietary fiber, antioxidants substances, and unsaturated and polyunsaturated fatty acids [5,6,7]. Due to its excellent nutritional value, buckwheat can be added to rice or corn flours to enhance their nutritional quality. Furthermore, buckwheat flour has showed superior ability to the other pseudo-cereals flours to produce gluten-free pasta, having a lower detrimental effect on firmness, cooking time and cooking loss [8].

The extrusion-cooking technique is one of the most appropriate technologies for the production of gluten-free pasta [9,10]. Moreover, modification of processing parameters (including moisture content, barrel temperature, and screw speed) during extrusion-cooking allows the use of several raw materials, and, thus, the production of pasta products with specific properties and functionality [11,12]. Some reports also suggest that extrusion-cooking does not degrade the valuable compounds found in raw plant materials (in buckwheat, mainly the phenolic acids). These, however, can be strongly affected by processing conditions [13,14].

However, to the authors’ knowledge, there are no studies about the production of gluten-free pasta based on rice and buckwheat flours by means of the extrusion-cooking technique. Therefore, we investigated the use of buckwheat flour to enrich rice-based pasta. The aims of this work were (a) to study the effect of extrusion-cooking parameters on cooking and textural properties of rice-buckwheat pasta, and (b) to evaluate the quality of pasta made using optimum parameters.

## 2. Materials and Methods

### 2.1. Raw Materials

Rice flour was obtained from Lubella Sp. z o. o. S.K. (Lublin, Poland). Buckwheat flour was bought from a local market and was sieved to obtain flour with particles sizes below 0.5 mm.

### 2.2. Pasta Processing

Gluten-free pasta was prepared using a mixture of rice flour and buckwheat flour with a ratio of 50:50. The blends were then moistened to a pre-determined level of moisture content (30, 33, and 36%) and allowed to stand for 30 min. A single screw extrusion-cooker type TS-45 (ZMCh Metalchem, Gliwice, Poland) equipped with an additional glycol cooling section before the die was used to produce pasta products at different barrel temperatures (80, 100, and 120 °C) in the first extruder section, 100 °C in the second section, and 70 °C in the cooling section. The pasta products were processed at 60, 80, and 100 rpm using the barrel configuration of length to screw diameter L/D = 18. Rice-buckwheat pasta products were shaped like spaghetti using a die with 12 × 0.80 mm round openings and cut for 30 cm pieces. After pre-drying for 5 min at room temperature, the pasta strands were distributed on perforated trays and then dried in an air dryer at 40 °C for 4 h. The dried pasta (moisture content below 12%) was cooled, packed in sealed plastic bags, and stored for further analysis.

### 2.3. Design of Experiments

Response surface methodology based on a Box-Behnken experimental design was applied to investigate the effect of extrusion-cooking parameters (*X*_1_ moisture content, *X*_2_ barrel temperature, and *X*_3_ screw speed) on the cooking and textural properties of the produced rice-buckwheat pasta. This design is a three-factor, three-level design. Each of the independent variables was coded at three levels (−1, 0, and +1). These correspond to low, intermediate, and high levels, respectively. The variables were applied as follows: 30, 33, and 36% for moisture content, 80, 100, and 120°C for barrel temperature and 60, 80, and 100 rpm for screw speed, respectively. The total number of experiments came out to be 15, with three replications at the center point (Table 1). The order of the experiments was fully randomized.

Responses included cooking loss (Y_1_), hardness (Y_2_), firmness (Y_3_), and stickiness (Y_4_). The optimal pasta samples were examined further for chemical composition, water absorption capacity, expansion ration, color, sensory attributes, and microstructure. A second-order polynomial model (Equation (1)) was defined to fit the responses:

Y = β_0_ + β_1_*X*_1_ + β_2_*X*_2_ + β_3_*X*_3_ + β_11_(*X*_1_)^2^ + β_22_(*X*_2_)^2^ + β_33_(*X*_3_)^2^ + β_12_*X*_1_*X*_2_ + β_13_*X*_1_*X*_3_ + β_23_*X*_2_*X*_3_ (1)

where *Y* is the response obtained by the fitted model and β_0_ is the model intercept. β_1_, β_2_, and β_3_ are the linear coefficients, β_12_, β_13_, and β_23_ are the interaction coefficients, and β_11_, β_22_, and β_33_ are the quadratic coefficients.

### 2.4. Optimization of Processing Parameters

To obtain optimal parameters for the production of rice-buckwheat pasta, an optimization method was applied using a desirability function. This approach consists of standardizing and converting each response (Y_i_) into individual desirability functions (*d_i_*) that vary from 0 (unacceptable response) to 1 (completely desirable response). The overall desirability (*D*) with *n* responses is calculated by applying the geometric mean (Equation (2)). Subsequently, an algorithm is applied to determine the levels of variables that maximize *D* [15]:

*D* = (*d*_1_ × *d*_2_ × *d*_3_ × *d*_*n*_)^1/*n*^ (2)

### 2.5. Quality Evaluation of Gluten-Free Pasta

#### 2.5.1. Chemical Composition

The tests of proximate chemical composition were performed for raw materials, as well as for optimum rice-buckwheat ground pasta (particles size below 300 µm). Protein (AACC 46-10), lipid (AACC 30-10), and ash (AACC 08-01) content were determined in triplicate according to AACC methods [16]. Dietary fiber was assessed according to 991.43 AOAC method [17] in two replications.

The total polyphenols content (TPC) was determined as follows. Aqueous-organic extracts were isolated as reported by Lisiecka et al. [18]. In a tube, 0.5 g of sample was placed with 5 mL of methanol/water 50:50 (*v*/*v*). The obtained extracts were stirred for 30 min using a rotator Multi RS-60 operating at 5000 rpm (Biosan, Riga, Latvia), then they were centrifuged (5000 rpm) for 10 min at 4 °C, using centrifuge MPW-352R (MPW, Warsaw, Poland). The extraction was performed in duplicate. The supernatants were recovered and directly assayed for TPC according to the Folin-Ciocalteau procedure [19]. In brief, 0.05 mL of extracts was oxidized by means of the 0.4 mL of Folin-Ciocalteau reagent (in a ratio of 1:5 with distilled water) in the presence of 0.05 mL of 50% methanol and 0.1 mL of H_2_O. The reaction was neutralized with 2 mL of 10% sodium carbonate. The absorbance was measured with a Helios Gamma Spectrophotometer (Thermo Electron Corporation, England) at 725 nm against a blank after 30 min of reaction at room temperature in a dark place. The TPC was expressed as gallic acid equivalent (GAE) in mg/g of dry weight (d.w.). Measurements were carried out twice.

#### 2.5.2. Minimal Preparation Time

Next, 10 g of pasta underwent hydration with 200 mL of hot water (~98 °C). The minimal preparation time (MPT) was achieved after the disappearance of the inner white core of the pasta strand when squeezing it between two Plexiglas plates [20]. The test was performed in triplicate.

#### 2.5.3. Water Absorption Capacity and Cooking Loss

After, 10 g of pasta were hydrated with 200 mL of hot water. After the corresponding MPT, the hydrated pasta was rinsed with tap water (20 °C) and drained for 5 min. Both hydrating and rinsing water were collected in a glass beaker and then evaporated in an air oven at 110 °C until constant weight was reached. Water absorption capacity (WAC) and cooking loss (CL) were calculated in triplicate using the following equations [21]:

(3) $$\mathrm{WAC}\;\left(g/100\;g\right)=\frac{\mathrm{weight}\;\mathrm{of}\;\mathrm{hydrated}\;\mathrm{pasta}-\mathrm{weight}\;\mathrm{of}\;\mathrm{dry}\;\mathrm{pasta}\;}{\mathrm{weight}\;\mathrm{of}\;\mathrm{dry}\;\mathrm{pasta}}\times \;100$$

(4) $$\mathrm{CL}\;\left(\%\right)=\frac{\mathrm{weight}\;\mathrm{of}\;\mathrm{dry}\;\mathrm{residue}}{\mathrm{weight}\;\mathrm{of}\;\mathrm{dry}\;\mathrm{pasta}}\times 100$$

#### 2.5.4. Texture Measurements

Hardness of dry pasta (in five replications) and firmness and stickiness of hydrated pasta (in two replications) were determined using a Zwick-Roell BDO-FB0.5 TH instrument (Zwick GmbH & Co. KG, Ulm, Germany) with the speed of 3.3 mm/s and the working head of 0.5 kN. Subsequently, TestXpert^®^10.11 software (Zwick GmbH & Co. KG, Ulm, Germany) was applied to calculate hardness (N) as the maximum cutting force using a Warner-Bratzler cutting knife (equipment supplied by Zwick), firmness (N) as the highest peak at first compression, and stickiness (mJ) as the energy needed to remove the working plate from the pasta surface after compression using an Ottawa Texture Measuring System cell (OTMS cell) (equipment supplied by Zwick) [11].

#### 2.5.5. Expansion Ratio

The expansion ratio (ER) was calculated by dividing the diameter of the pasta strand (average of 10 replications) by the die diameter.

#### 2.5.6. Color

The color of dry and hydrated pasta was evaluated using Lovibond CAM-System 500 Imaging Colorimeter (The Tintometer Ltd., Amesbury, UK) in 10 replications. Results are expressed using CIE-Lab color scale. Herein, L* values measure lightness (0 = black–100 = white), a* values measure redness (positive values) and greenness (negative values), and b* values measure yellowness (positive values) and blueness (negative values).

#### 2.5.7. Sensory Evaluation

Rice-buckwheat pasta (100 g) was hydrated in hot water for the corresponding MPT, drained and served after 5 min on white plates with no suggestive information to a panel of 15 untrained members educated with requirements for pasta products. Panelists evaluated appearance, color, flavor, taste, and stickiness of pasta by applying a five-point scale (1 = poor, 5 = good) as described by Wójtowicz and Mościcki [20]. The same panel assessed the overall acceptability of a pasta sample using a verbal nine-point hedonic scale (1 = dislike extremely, 5 = neither like nor dislike, 9 = like extremely). Before the sensory evaluation, the panelists were informed of how to rate each attribute from 1 to 5 and how to evaluate the overall acceptability from dislike extremely to like extremely. The pasta was considered acceptable if its overall acceptability mean score was above 5 [22]. The tests were carried out in a laboratory space with appropriate ventilation, neutral background, proper lighting, and minimal traffic, which was free from distractions, noise, and odors. Informed consent was obtained from each participant.

#### 2.5.8. Microstructure

The microstructure of dry and hydrated rice-buckwheat pasta was characterized using scanning electron microscopy (SEM). The hydrated pasta was lyophilized before characterization. Pasta samples of 5 mm were mounted on carbon discs and sputter-coated with gold in a vacuum sublimatorK-550X (Emitech, RC, Ashford, England). The scanning electron microscope VEGA LMU (Tescan, Warrendale, PA, USA) operating at the accelerating voltage of 30 kV was used to characterize the cross-section of samples at different magnifications (200× and 600× for sample surfaces and 100×, 600×, and 2000× for sample cross-sections).

### 2.6. Statistical Analysis

Statistical analysis was performed using JMP software version 7.0 (SAS Institute Inc., Cary, NC, USA). The experimental data were evaluated by means of multiple regression analysis and one-way analysis of variance (ANOVA). Validation of the model was expressed by the coefficient of determination (*R*^2^) and its statistical significance was ascertained by a *F*-test. Models were considered significant at *p* values <0.05. Statistica software version 10 (StatSoft. Inc., Tulsa, OK, USA) was applied to perform statistical differences between mean values at *p* < 0.05 using one-way analysis of variance (ANOVA), followed by the Tukey’s honestly significant difference (HSD) post hoc test.

## 3. Results and Discussion

### 3.1. Model Adequacy

The regression model *F*-test values were 23.78, 9.62, 27.26, and 9.47 for cooking loss, hardness, firmness, and stickiness, respectively (Table 2). Here, *p*-values lower than 0.05 indicate that the models and the associated terms are statistically significant. The goodness of fit for all models was further confirmed by a satisfactory value of coefficients of determination (*R*^2^) (these were found to be between 0.94 and 0.98).

### 3.2. Effect of Processing Parameters on Cooking Loss

Table 3 presents the effects of extrusion-cooking parameters on cooking loss (CL) and textural properties of rice-buckwheat pasta processed under various conditions.

**Figure 1:**
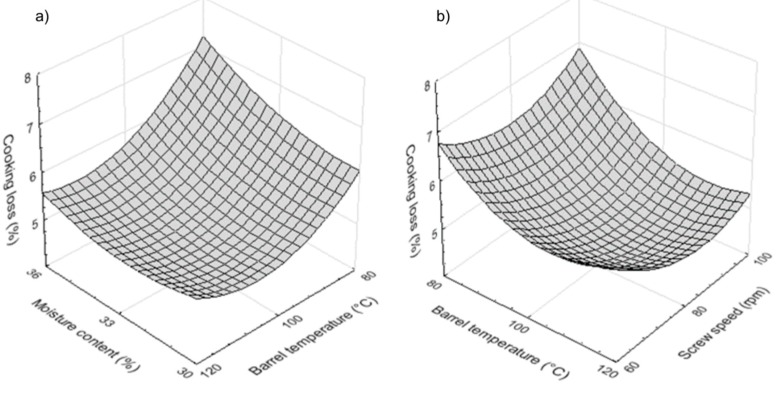
Effect of (**a**) moisture content and barrel temperature and (**b**) barrel temperature and screw speed on cooking loss of rice-buckwheat pasta.

As indicated, moisture content displays a significant positive effect (*p* < 0.01) on CL. This increased with the increase of dough moisture content (Figure 1a), especially when low barrel temperature was applied during pasta processing. The effect could be the result of a higher moisture level, as this is known to lead to the development of sticky pasta with low mechanical strength and, hence, higher solid losses during hydration. Furthermore, the pressure inside the extruder decreases as dough moisture content increases, causing faster feed flow which leads to an unstable texture after drying and, therefore, higher cooking loss [23]. Similar findings have been reported in the literature for gluten-free pasta made from various raw materials [11,24,25,26].

Barrel temperature had a great negative significant effect (*p* < 0.001) on CL. This parameter decreased as barrel temperature increased; in this case, the effect of screw speed was less important (Figure 1b). Giménez et al. [25] and Wang et al. [27] reported similar observations for gluten-free pasta produced by extrusion-cooking. Accordingly, the melt viscosity of starch decreases as barrel temperature increases, causing lower friction stress in the extruder, which leads to lower molecular degradation and therefore lower cooking loss [27]. Moreover, CL was positively related to stickiness (*r* = 0.73, *p* < 0.05).

### 3.3. Effect of Processing Parameters on Hardness, Firmness and Stickiness

**Figure 2:**
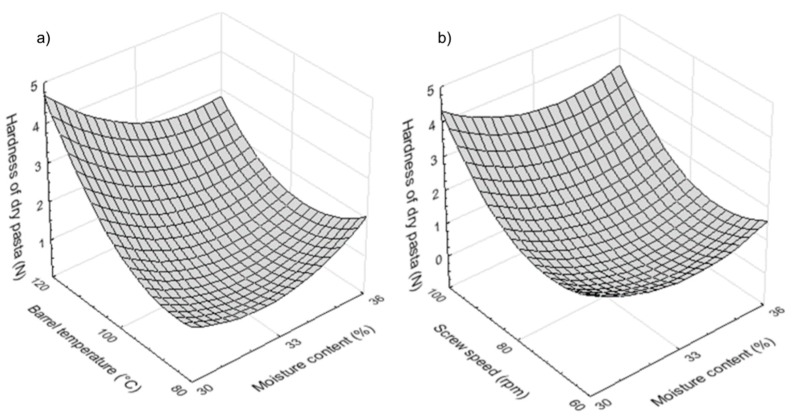
Effect of (**a**) moisture content and barrel temperature and (**b**) screw speed and moisture content on hardness of dry rice-buckwheat pasta.

It seems clear from the estimate regression coefficients presented in Table 2 that hardness of dry pasta was affected significantly (*p* < 0.01) by barrel temperature and screw speed. The positive estimate coefficients of barrel temperature and screw speed reveal that hardness of dry pasta increased with increasing barrel temperature (Figure 2a) and screw speed (Figure 2b). Wójtowicz [28], too, found an increase in hardness of dry buckwheat pasta with an increase of screw speed during processing. Hardness of dry pasta therein, was positively correlated with firmness (*r* = 0.76, *p* < 0.05).

**Figure 3:**
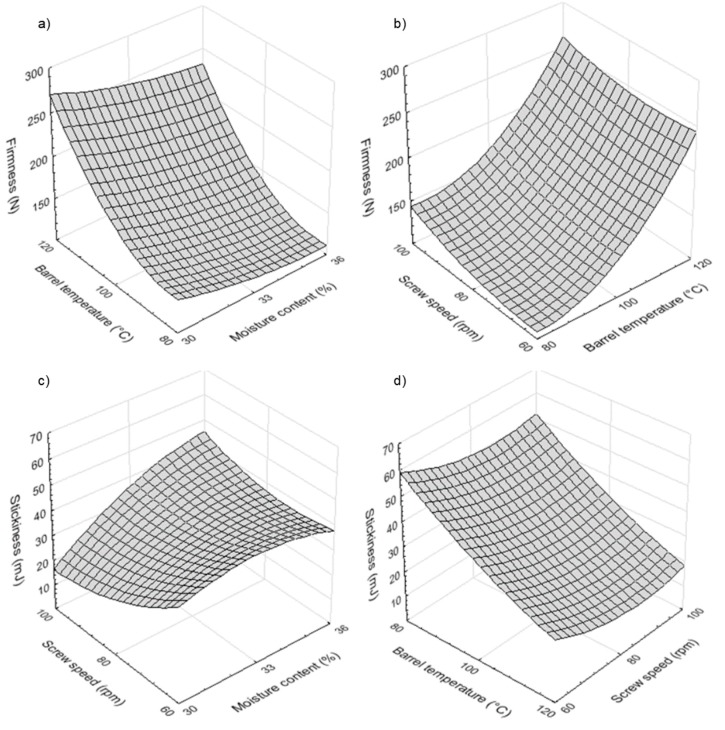
Effect of moisture content, barrel temperature and screw speed on (**a**,**b**) firmness and (**c**,**d**) stickiness of hydrated rice-buckwheat pasta.

Firmness of hydrated pasta was affected by all processing parameters. Moisture content showed a negative significant effect (*p* < 0.05), while barrel temperature and screw speed had a positive significant effect (*p* < 0.001 and *p* < 0.05, respectively). Overall, firmness of rice-buckwheat pasta increased when dough moisture content decreased (Figure 3a). Similar observations were reported by Wójtowicz [28] for buckwheat pasta. At higher moisture content, shear force decreases and, therefore, the gelatinization degree decreases, causing low firmer texture [23].

In contrast, firmness of hydrated pasta increased when barrel temperature and screw speed increased (Figure 3b). Wang et al. [27] reported similar observations for pea starch noodles. The increase in temperature and screw speed has been reported to cause an increase of gelatinization, thus producing pasta with improved strength and firmer texture [23]. Of note, firmness was negatively correlated with stickiness (*r* = −0.84, *p* < 0.05).

Stickiness was affected positively by the initial moisture content of raw materials (*p* < 0.05) and negatively by the barrel temperature (*p* < 0.001). Stickiness of hydrated pasta decreased as barrel temperature increased (Figure 3d) and increased as moisture content increased (Figure 3c). Similar observations were reported by Bouasla et al. [11] for gluten-free pasta products. In the extrudates processed at high level of moisture content, the viscosity of starch inside the extruder barrel is relatively low, which reduces the effect of screw speed on shearing stress, resulting in less degradation of amylose and amylopectine [11]. Wang et al. [27] reported that stickiness of pasta is affected by amylose released onto the surface of pasta strand during cooking and by pasta surface structure.

### 3.4. Optimal Parameters for Rice-Buckwheat Pasta-Making

The optimum levels of processing parameters were obtained by applying the desirability function of minimizing cooking loss and stickiness, and maximizing the hardness of dry pasta and the firmness of hydrated pasta. The optimal conditions were 30% for moisture content, 120 °C for barrel temperature, and 80 rpm for screw speed, with a desirability function of 0.86.

### 3.5. Characteristics of Optimum Rice-Buckwheat Pasta

#### 3.5.1. Chemical Composition

On a dry basis, buckwheat flour was characterized by significantly higher protein (10.55%), ash (1.58%), fiber (4.57%), and TPC (1.85 mg GAE/g) contents than rice flour (7.72%, 1.37%, 1.31%, and 0.04 mg GAE/g respectively) (Table 4). Bouasla et al. reported similar observations for rice-buckwheat pasta composition (protein 9.78%, ash 1.07%, and fiber 5.21%) if compared to rice pasta (protein 8.25%; ash 0.50%, and fiber 3.21%) [21]. The high amount of fiber recorded in rice-buckwheat pasta would be beneficial for persons with celiac disease since, as noted by Stojceska et al. [29], they have low dietary fiber intake. The increased fiber in extruded products could be due to the effect of insoluble fibrous fractions being created because of the transformations occurring during the extrusion-cooking, which may have an effect on the formation of resistant starch [30].

In our experiment, incorporation of buckwheat flour led to an increase of TPC in rice-buckwheat pasta (0.40 mg GAE/g) 10 times more than that of rice flour (0.04 mg GAE/g). Phenolic acids (especially of protocatechuic acid and the cinnamic series) and flavonoids (especially quercetin and rutin) make up most of the polyphenol classes found in buckwheat [31]. The extent and the mechanism of the protective action of phenolic compounds is due to their anti-inflammatory, anticarcinogenic, antibacterial, and antiviral activity in the human body, as well as their high antioxidant capacity [18,32]. Therefore, the presence of phenolic compounds in gluten-free pasta would also be beneficial for persons with celiac disease.

In addition, very low-fat content (0.16%) has been noticed for rice-buckwheat pasta, as compared to the separate raw materials (2.30% and 2.04% for rice flour and buckwheat flour, respectively). Similar observations have been reported for wheat-based and rice-based pasta manufactured by extrusion-cooking [20,21]. This phenomenon is related to the formation of amylose-lipids complexes during pasta processing by extrusion-cooking, causing a decrease in lipids extractability [33].

#### 3.5.2. Cooking, Physical, and Textural Properties of Rice-Buckwheat Pasta

Table 5 shows the characteristics of rice-buckwheat pasta produced at the optimal processing parameters, in comparison to rice pasta, as reported in our previous study [21].

Hot water hydration for 8.5 min was sufficient for rice-buckwheat pasta to have the proper consistency. This differs from conventional cooking. The comparison of our results with previous studies using the same method of hydration in hot water revealed that rice-buckwheat pasta had a MPT slightly higher than the time reported by Bouasla et al. [21] for rice pasta (8 min) and much higher than reported by Wójtowicz and Mościcki [20] for common wheat pasta (5.5 min).

Water absorption capacity of rice-buckwheat pasta (237.77 g/100g) was also significantly higher than that reported for rice pasta (181.53 g/100 g). This effect could be explained by the higher water-binding capacity of buckwheat starch [34], as well as by its high content of fiber, which has the ability to absorb water very efficiently. The capacity, as noted by Wójtowicz and Mościcki [20], is, however, slightly lower than that reported for precooked common wheat pasta (260 g/100 g) and demonstrates the presence of gluten proteins.

Rice-buckwheat pasta had significantly higher CL (5.23%) than rice pasta (3.57%). This increase in CL could be related to the presence of fiber, as this weakens the starch network in both gluten-free matrices and gluten-based matrices [21]. However, the obtained value indicated good quality of rice-buckwheat pasta, since 10% of cooking loss was reported as a quality limit for pasta [35]. Our work shows that the structure of precooked products was able to preserve the pasta integrity during hot water hydration and, therefore, to minimize cooking loss. Indeed, during extrusion-cooking, heat, mechanical shearing, and pressure contributed in the formation of a homogenous and compact starch-protein matrix. This was confirmed by the pasta microstructure assessment (Figure 5c). Similar findings have been reported for gluten-free pasta in previous studies [11,20,21]. In addition, Vallons and Arendt [36] reported that pressure-thermal treatment makes buckwheat starch granules more resistant to swelling and disintegration under the influence of additional heat, due to the gelatinization of buckwheat starch between 70 and 75 °C.

Our work showed that the expansion ratio of rice-buckwheat pasta (1.21) was significantly lower than that of rice pasta (1.55). This may be due to the low amount of total starch and high amount of protein and fiber in buckwheat flour, which limits the expansion of pasta products [21]. The increase of dough viscosity inside the extruder barrel induced by the increase of protein and fiber content involves longer residence time and higher mechanical shearing forces, which may cause a higher extent of starch molecular degradation, leading to a low expansion ratio [27]. Yet, as observed by Wójtowicz and Mościcki [37], the effect of high fiber content on extrudates may also limit the expansion of extruded products for common wheat pasta supplemented with bran addition or breakfast cereals based on wholegrain wheat flour [38].

Pasta color is considered an essential parameter for evaluating pasta quality [39]. Both dry and hydrated pasta products contain buckwheat flour. This is characterized as having lower lightness, less green tint, and more intensive yellowness than rice pasta (Table 3). These results can be related to the darker color and the presence of carotenoid pigments in buckwheat flour.

Dry rice-buckwheat pasta showed significant lower hardness (4.05 N) than rice pasta (12.12 N). This effect could be related to the lower diameter of rice-buckwheat pasta, compared to rice pasta, and to the presence of fiber fractions from buckwheat flour. These fiber fractions could weaken pasta structure by the formation of discontinuities or cracks inside the pasta strand [39].

Adding buckwheat flour into the pasta recipe increased the firmness of the hydrated pasta. Firmness values differed significantly, with values of 199.5 N for rice pasta and 271.0 N for rice-buckwheat pasta. This can be explained by the higher protein content in rice-buckwheat pasta, compared to rice pasta and the formation of more firm structure after hot water hydration.

The incorporation of buckwheat flour increased significantly the stickiness of hydrated pasta (2.48 mJ and 9.22 mJ for rice pasta and rice-buckwheat pasta, respectively). This increase in pasta stickiness could be the result of higher fiber content and/or higher amount of compounds leached from the surface of rice-buckwheat pasta during hot water hydration, as confirmed by higher cooking loss compared to rice pasta. Alamprese et al. [7] showed that replacement of wheat flour by buckwheat in lasagna had an effect on lower break load and break strain values of cooked products if the amount of buckwheat flour in the recipe increased.

#### 3.5.3. Sensory Attributes

For all of the sensory attributes of hydrated pasta, rice-buckwheat products received higher scores than rice pasta (Table 6). When appearance, color, flavor, taste, and stickiness were evaluated in a 5-point scale, all the results were above 4.0 for rice-buckwheat pasta. This indicates that the optimized pasta is of very good quality. Moreover, while the results of stickiness performed by an instrumental assessment were higher for rice-buckwheat pasta, in a sensory test, consumers did not confirm the higher stickiness of supplemented pasta, because the specific taste and consistency of rice-buckwheat pasta and increased stickiness had no negative effect on sensory feelings. In addition, for overall acceptability, and utilizing a 9-point hedonic scale, rice-buckwheat pasta received higher scores (7.27) than rice pasta (6.53).

The replacement of 50% of starchy components by pseudo-cereals such as buckwheat in bread [4] or 25% of a recipe in gluten-free pasta [40] can improve nutrients content, including proteins and minerals (calcium, magnesium, zinc, and iron). Our work and previous studies show that application of buckwheat flour has no negative effect on the sensory profile, especially for pasta surface smoothness, odor, and overall acceptability [40]. Moreover, beyond enhanced nutritional value, buckwheat also has a specific taste that can have a positive effect on the sensory profile of gluten-free products. This notion is confirmed by our results.

#### 3.5.4. Microstructure

**Figure 4:**
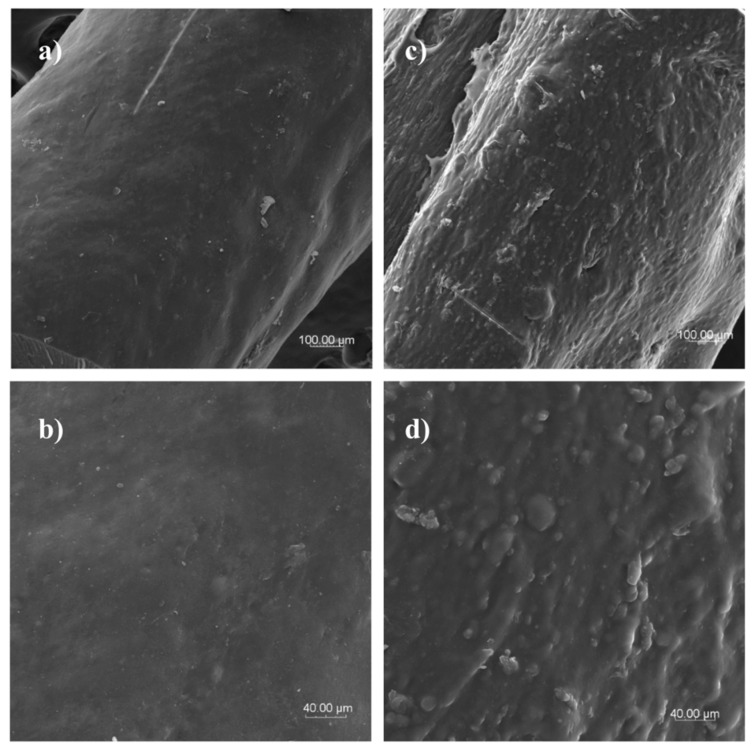
Surface of dry (**a**,**b**) and hydrated (**c**,**d**) rice-buckwheat pasta at different magnifications (200× for **a**,**c**; 600× for **b**,**d**).

**Figure 5:**
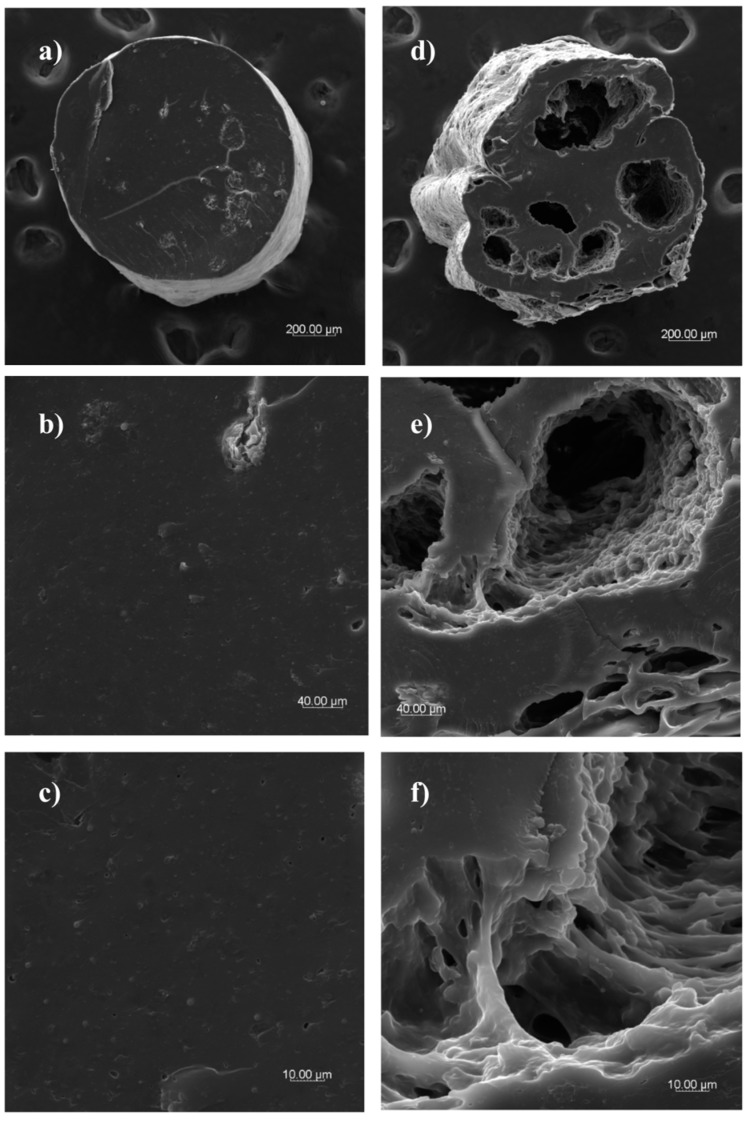
Cross-section of dry (**a**–**c**) and hydrated (**d**–**f**) rice-buckwheat pasta (magnification 100×, 600×, 2000×, respectively).

The microstructure of dry and hydrated optimum rice-buckwheat pasta was observed with a scanning electron microscopy. Herein, dry rice-buckwheat pasta demonstrated a smooth surface (Figure 4a,b). After hydration in hot water, the surface of pasta presented a slightly rough surface probably due to the leaching of unbounded compounds to hot water from the pasta surface (Figure 4c). At high magnification, the surface of pasta highlighted the presence of melted materials (Figure 4d). When the processing temperature of optimum rice-buckwheat pasta was found to be 120 °C, with the initial moisture of 30% and screw speed at 80 rpm during processing, a proper and stable structure was formed, both for the surface (Figure 4b) and cross-section (Figure 5c) of the dry pasta. This compact structure was reached due to the integrated effect of heating and shearing of rice-buckwheat blends during extrusion-cooking [10]. Past studies reinforce this notion. Vallons and Arendt [36] undertook SEM imaging for pressure-thermal treated buckwheat starch and showed a complete breakdown of the granular structure after treatment above 65 °C, with the starch concentration in a treated solution being 25%.

A homogenous and compact inside structure was noted during the observation of the cross-sectional microstructure of dry pasta (Figure 5a–c). This structure is due to the effect of extrusion-cooking conditions (moisture, high temperature, and mechanical shearing forces). This induces starch gelatinization and retrogradation, and, thus, a coherent structure [11,12,21]. Under high magnification (Figure 5b), only a few starch granules were observed; these were surrounded by a melted uniform matrix formed as a result of starch gelatinization during processing. The cross-section of hydrated pasta is characterized by compact structure with visible empty spaces inside the pasta tread that resulted from the leaching of components during hot water hydration. Moreover, under high magnification, the external parts of the pasta show a partly porous structure that is due to migration of hot water inside pasta (Figure 5d). Such effect supports the low values of cooking loss found in this study. Hydrated optimum rice-buckwheat pasta observed at high magnification (Figure 5e,f) showed diversified internal structure based on the visible homogenous matrix, with several spaces filled with a melted starch-protein matrix, with long bounded complexes keeping the structure compact when the pasta underwent hot water hydration. This was confirmed by the high firmness results of the tested pasta.

## 4. Conclusions

The effect of extrusion-cooking parameters on the quality of rice-buckwheat gluten-free pasta was studied using a Box-Behnken design. The results indicated that moisture content, barrel temperature, and screw speed had different effects on the cooking and textural properties of gluten-free pasta. Barrel temperature significantly affected all tested properties. An increase in barrel temperature induced a decrease in cooking loss and stickiness and an increase in hardness and firmness, while the reverse effect was observed for moisture content except for hardness. Screw speed also showed a significant positive effect, albeit only on hardness and firmness.

In our work, an optimum gluten-free pasta was produced at 30% of moisture content, 120 °C for temperature, and 80 rpm for screw speed and was then evaluated for its characteristics. This optimum rice-buckwheat pasta showed good quality, specifically for high nutritional composition, low cooking loss and stickiness, and acceptable score for all sensory attributes and overall acceptability, with homogeneous and compact microstructure. Based on the obtained results, gluten-free rice-buckwheat pasta would constitute as a good alternative for persons afflicted with celiac disease.

## Figures and Tables

**Table 1:** Box-Behnken experimental design with coded and real values of extrusion-cooking variables.

	Coded Values			Real Values		
Run	X_1_	X_2_	X_3_	Moisture Content (%)	Barrel Temperature (°C)	Screw Speed (rpm)
1	–1	–1	0	30	80	80
2	+1	–1	0	36	80	80
3	–1	+1	0	30	120	80
4	+1	+1	0	36	120	80
5	–1	0	–1	30	100	60
6	+1	0	–1	36	100	60
7	–1	0	+1	30	100	100
8	+1	0	+1	36	100	100
9	0	–1	–1	33	80	60
10	0	+1	–1	33	120	60
11	0	−1	+1	33	80	100
12	0	+1	+1	33	120	100
13	0	0	0	33	100	80
14	0	0	0	33	100	80
15	0	0	0	33	100	80

**Table 2:** *F*-values and coefficient of determination for different responses.

Responses	Model		
	*F*-value	*p*-value	*R* ^2^
Cooking loss (%)	23.7806	0.0014*	0.98
Hardness (N)	9.6248	0.0113*	0.95
Firmness (N)	27.260	0.0010**	0.98
Stickiness (mJ)	9.4727	0.0117*	0.94

* Significant at *p* < 0.05, ** Significant at *p* < 0.01.

**Table 3:** Significant estimates of the regression coefficients of the model for the different responses.

Responses	Estimates of Regression Coefficients		
	Moisture content (%)	Barrel Temperature (°C)	Screw Speed (rpm)
Cooking loss (%)	0.3275 **	−0.595 ***	*ns*
Hardness (N)	*ns*	1.01375 **	1.055 **
Firmness (N)	−16.6875 *	63.3125 ***	13.125 *
Stickiness (mJ)	7.135 *	−16.9875 ***	*ns*

* Significant at *p* < 0.05, ** Significant at *p* < 0.01, *** Significant at *p* < 0.001, *ns* indicated coefficient does not contribute significantly in the model.

**Table 4:** Chemical composition (d.w.) of flours and gluten-free optimum pasta.

Samples	Protein (%)	Fat (%)	Ash (%)	Fiber (%)	TPC (mg GAE/g)
Rice flour	7.72 ± 0.004 ^a^	2.30 ± 0.01 ^c^	1.37 ± 0.02 ^b^	1.31 ± 0.01 ^a^	0.04 ± 0.01 ^a^
Buckwheat flour	10.55 ± 0.01 ^c^	2.04 ± 0.01 ^b^	1.58 ± 0.01 ^c^	4.57 ± 0.01 ^b^	1.85 ± 0.20 ^b^
Rice-buckwheat optimum pasta	9.78 ± 0.01 ^b^	0.16 ± 0.01 ^a^	1.07 ± 0.01 ^a^	5.21 ± 0.01 ^c^	0.40 ± 0.02 ^a^

Means ± standard deviations; ^a–c^: means indicated with similar letters in columns do not differ significantly at 0.05. TPC: total polyphenols content.

**Table 5:** Characteristics of Optimum Rice-Buckwheat Pasta in Comparison to Rice Pasta.

	Rice-Buckwheat Pasta	Rice Pasta [21]
Minimal preparation time (min)	8.5 ± 0.01 ^a^	8.0 ± 0.01 ^a^
Water absorption capacity (g/100g)	237.77 ± 0.81 ^b^	181.53 ± 6.68 ^a^
Cooking loss (%)	5.23 ± 0.12 ^b^	3.57 ± 0.30 ^a^
Expansion ratio (-)	1.21 ± 0.05 ^a^	1.55 ± 0.03 ^b^
Color of dry pasta		
L*	42.99 ± 2.11 ^a^	72.29 ± 0.81 ^b^
a*	0.48 ± 1.28 ^b^	−4.00 ± 0.33 ^a^
b*	32.34 ± 3.03 ^b^	29.25 ± 1.22 ^a^
Color of hydrated pasta		
L*	65.67 ± 2.00 ^a^	79.07 ± 1.44 ^b^
a*	−2.27 ± 1.88 ^b^	−5.25 ± 0.63 ^a^
b*	16.49 ± 1.19 ^b^	13.71 ± 0.82 ^a^
Hardness (N)	4.05 ± 0.21 ^a^	12.12 ± 1.41 ^b^
Firmness (N)	271.0 ± 9.90 ^b^	199.5 ± 0.71 ^a^
Stickiness (mJ)	9.22 ± 0.21 ^b^	2.48 ± 0.13 ^a^

Means ± standard deviations; ^a-b^: means indicated with similar letters in rows do not differ significantly at 0.05; L*: lightness, a*: red-green balance, b*: yellow-blue balance.

**Table 6:** Sensory attributes of optimum rice-buckwheat pasta in comparison to rice pasta.

Attributes	Rice-Buckwheat Pasta	Rice Pasta [21]
Appearance	4.40 ± 0.63 ^a^	4.00 ± 1.13 ^a^
Color	4.00 ± 1.13 ^b^	3.27 ± 1.16 ^a^
Flavor	4.40 ± 0.99 ^b^	3.93 ± 1.03 ^a^
Taste	4.33 ± 0.72 ^a^	3.93 ± 1.03 ^a^
Stickiness	4.87 ± 0.35 ^b^	4.13 ± 0.92 ^a^
Overall acceptability	7.27 ± 1.28 ^b^	6.53 ± 1.51 ^a^

Means ± standard deviations; ^a-b^: means indicated with similar letters in rows do not differ significantly at 0.05.
